# Editorial: Dietary exposures to environmental pollutants: integrated multimedia perspectives

**DOI:** 10.3389/fpubh.2023.1283783

**Published:** 2023-11-01

**Authors:** Peng Gao, Yuxia Ma, Gangqiang Ding, Qun Xu

**Affiliations:** ^1^Department of Environmental and Occupational Health, Department of Civil and Environmental Engineering, University of Pittsburgh, Pittsburgh, PA, United States; ^2^Department of Nutrition and Food Hygiene, School of Public Health, Hebei Medical University, Hebei Province Key Laboratory of Environment and Human Health, Shijiazhuang, China; ^3^National Institute for Nutrition and Health, Chinese Center for Disease Control and Prevention, Beijing, China; ^4^Department of Epidemiology and Biostatistics, Institute of Basic Medical Sciences Chinese Academy of Medical Sciences, School of Basic Medicine Peking Union Medical College, Beijing, China

**Keywords:** environmental pollutants, dietary exposures, public health, mixture effect, environmental health, exposome, emerging contaminants, chronic diseases - prevention and control

The global challenge of understanding the intricate links between dietary exposures and environmental pollutants is crucial for public health and necessitates a comprehensive and multifaceted approach. Environmental pollutants, broadly encompassing chemicals and particles present in our air, water, soil, and food, have long been a cause of concern for their potential adverse effects on human health. Among the multiple pathways of exposure to these pollutants, diet is particularly significant as it is a primary route through which humans are exposed to various environmental contaminants. This is because pollutants can accumulate in the food chain, leading to higher concentrations in the foods consumed by humans. Moreover, the interaction between diet and environmental pollutants is complex and multifactorial, involving various biological, environmental, and lifestyle factors. Therefore, a thorough understanding of the relationships between dietary exposures and environmental pollutants is essential for developing effective strategies to minimize the risk and impact of these pollutants on human health. This Research Topic, entitled “*Dietary exposures to environmental pollutants: integrated multimedia perspectives*,” includes 15 articles that delve into various aspects of dietary exposures and their associations with environmental pollutants, providing integrated perspectives on this critical subject.

Several articles in this issue address the broader environmental context. Azanaw et al. conducted a multilevel analysis of improved drinking water sources and sanitation facilities in Ethiopia, which is fundamental to public health. The study revealed that while access to improved water sources is moderate, access to improved sanitation was significantly lower, underlining the need for significant improvements in providing access to improved water sources and sanitation facilities in Ethiopia. Additionally, He et al. investigated the spatial and temporal patterns, exposure risks, and drivers of surface ozone (O_3_) pollution in China, providing a broader perspective on air pollution and its effects. The study revealed a significant increase in O_3_ concentrations and associated premature deaths from respiratory diseases, emphasizing the need for region-specific O_3_ control policies. These two articles highlighted the broader environmental context of dietary exposures and environmental pollutants and called for a comprehensive and targeted approach to address the fundamental public health issues related to environmental pollutants.

Other studies explore the impacts of specific pollutants. Jhuang et al. investigated the latency period between aristolochic acid (AA) exposure and the development of upper urinary tract urothelial carcinoma (UTUC) in a Taiwanese cohort. The study highlighted a decreased risk of UTUC after the ban on AA in Taiwan, particularly among middle-aged women with moderate to high AA exposure and men with moderate AA exposure. The findings underscored that the latency period of UTUC varies with age, AA exposure dose, and gender. de Morais Valentim et al. conducted a multiscale analysis of pesticide residues in food in Brazil, estimating dietary cancer risks and associated mechanisms. The study provided a critical analysis of the pesticide scenario in Brazil from geographical, political, and public health perspectives, highlighting human rights violations due to food and water contamination. Additionally, Wang G.-X. et al. examined the association between urinary nickel and obesity status in adults using data from the National Health and Nutrition Examination Surveys. The study found a correlation between urinary nickel levels and body mass index and waist circumference in adult males, suggesting the need for obese men to reduce nickel exposure. These articles explored the impacts of specific pollutants, including aristolochic acid, pesticide residues, and urinary nickel, on various health outcomes. The findings highlighted the need for targeted interventions, such as the ban on aristolochic acid in Taiwan, and a comprehensive analysis of pesticide residues in food in Brazil to address human rights violations due to food and water contamination.

Several articles explore the relationship between environmental pollutants and specific health outcomes. Wei et al. conducted an age-period-cohort analysis to explore trends in cardiovascular disease (CVD) mortality attributable to non-optimal temperatures in China. Despite reductions in CVD mortality attributable to non-optimal temperatures, the study revealed a steady increase in ischemic heart disease mortality and the burden from high temperatures, calling for more strategies to protect human health from climate change impacts. Xiao et al. conducted a systematic review and meta-analysis to explore the association between per- and polyfluoroalkyl substances (PFAS) and the risk of hypertension. The study found that higher levels of certain PFAS, namely perfluorooctane sulfonate, perfluorooctanoic acid, and perfluorohexane sulfonate, were correlated with an increased risk of hypertension. Moreover, Zhang Y. et al. performed a bibliometric analysis of the association between drinking water pollution and bladder cancer. The study provided an overall and intuitive understanding of this topic in recent years, highlighting key research hotspots and revealing trends for further in-depth study in the future. These articles discussed the relationship between environmental pollutants and specific health outcomes, including cardiovascular disease mortality, hypertension, and bladder cancer. The findings suggested the need for strategies to protect human health from the impacts of climate change, a deeper understanding of the association between PFAS and hypertension, and a comprehensive analysis of the association between drinking water pollution and bladder cancer.

A group of articles delves into more specific dietary exposures. Jiao et al. investigated the association between egg intake and cardiometabolic factors (CMFs) in Chinese adults. They observed a U-shaped association between egg intake and CMFs, indicating that moderate egg consumption might lower the risk of central obesity, elevated triglycerides, decreased HDL-C, and elevated plasma glucose, whereas higher egg intake did not show a significant association with CMFs. Zhang S. et al. evaluated the relationship between body fat percentage (BF%) and the risk of type 2 diabetes (T2D) in Chinese adults. Analyzing data from 5,595 adults aged 18-65, they found that the incident risk of T2D significantly increased over specific levels of total and trunk BF% in both Chinese males and females. Their findings suggest that optimal BF% cut-off values may be valuable for clinical applications in the prevention and treatment of T2D in China. Additionally, Wang G. et al. explored the association between intestinal microbiota markers, dietary patterns, and T2D in Chinese patients. The study revealed significant associations between dietary intake patterns and gut flora, highlighting the crucial role of gut microbiota in linking dietary intake and the etiology of T2D. Another study by Wang N. et al. examined the role of rare earth elements (REEs) and dietary intake in the development of tongue cancer in southeast China. The findings suggest that some REEs interact with food intake to influence tongue cancer risk, while others act as mediators. These articles delved into specific dietary exposures and their association with health outcomes, including cardiometabolic factors, T2D, and tongue cancer. The findings suggest that moderate egg consumption may lower the risk of central obesity and other cardiometabolic factors, while optimal body fat percentage cut-off values may be valuable for the prevention and treatment of T2D. Additionally, the association between dietary intake patterns, gut flora, and T2D, and the interaction between REEs and food intake in the development of tongue cancer, highlight the crucial role of diet in disease development.

The final set of articles focuses on emerging contaminants and their health effects. Chen X. et al. discussed the adverse health effects of emerging contaminants on inflammatory bowel disease (IBD). The authors highlighted that emerging contaminants, such as microplastics, endocrine-disrupting chemicals, chemical herbicides, heavy metals, and persisting organic pollutants, may lead to many chronic diseases, including IBD. The article underscores the need to understand the impact of these new emerging contaminants on IBD and minimize their exposures to lower the future incidence of IBD. Liu et al. presented a nested case-control study examining the relationship between urinary selenium levels and the risk of gestational diabetes mellitus (GDM). The study found a significant negative association between urinary selenium and the risk of GDM, with this association varying depending on the fetal sex. The findings suggest that lower urinary selenium levels are associated with a higher risk of GDM, particularly among pregnant women with female fetuses. In another study, Chen Y. et al. investigated the role of endocrine-disrupting chemicals (EDCs) as a promoter of non-alcoholic fatty liver disease (NAFLD). The authors summarized the major EDCs contributing to the growing burden of NAFLD and aimed to raise public awareness regarding the hazards posed by EDCs to reduce the incidence of NAFLD. Overall, these articles focused on the adverse health effects of emerging contaminants on specific diseases like IBD, GDM, and NAFLD. The findings underscored the need to understand the impact of emerging contaminants on chronic diseases and to minimize exposures to lower the incidence of them.

Collectively, the articles in this Research Topic offer an integrative framework for dissecting the nuanced interplay between dietary exposures, environmental pollutants, and a range of health outcomes. The research presented not only deepens our understanding of these complex relationships but also serves as a crucial foundation for developing evidence-based public health policies and interventions. Specifically, these findings underscore the pressing need for targeted strategies that can mitigate the harmful impacts of environmental pollutants through informed dietary choices and lifestyle modifications. As our understanding evolves, it remains crucial to perpetually scrutinize these interconnections and their mechanistic underpinnings, intending to formulate more refined and effective preventative and management approaches for the myriad health risks associated with environmental exposures. In light of the comprehensive insights offered, the editors are optimistic that this Research Topic will engross the readership of Frontiers in Public Health and are confident that this topic will act as a catalyst for future research endeavors, encouraging multidisciplinary approaches to explore the multifaceted dimensions of dietary exposures to environmental pollutants.

## Author contributions

PG: Conceptualization, Writing – original draft, Writing – review & editing. YM: Writing – review & editing. GD: Writing – review & editing. QX: Writing – review & editing.

